# Antimicrobial susceptibility patterns of bacteria isolated from patients with ear discharge in Jimma Town, Southwest, Ethiopia

**DOI:** 10.1186/s12901-018-0065-0

**Published:** 2018-12-04

**Authors:** Kasahun Gorems, Getenet Beyene, Melkamu Berhane, Zeleke Mekonnen

**Affiliations:** 1 School of Medical Laboratory Sciences, Institute of Health, Jimma University, P.O. Box 378, Jimma, Ethiopia; 2 Department of Pediatrics and Child Health, Institute of Health, Jimma University, Jimma, Ethiopia

**Keywords:** Otitis media, Antimicrobial, Susceptibility, Jimma, Ethiopia

## Abstract

**Background:**

Otitis media is among the leading causes of childhood illnesses although it can also affect the adults resulting in frequent physician visits, drug prescription and a key contributor to antibiotic resistance. The aim of this study was to determine the risk factors, bacterial profile, and the antimicrobial susceptibility pattern of the isolates from patients with discharging ears which clinically equates to draining otitis media in developing countries with limited medical resources such as otoscope.

**Methods:**

A prospective cross-sectional study was conducted on 173 patients with draining otitis media. The ear discharge specimens were collected and analyzed by standard microbial techniques. The antibiotic susceptibility profiles were determined for 19 different antibiotics by the standard disk diffusion method. Data was analyzed by SPSS version 22 and the *P* value of less than 0.05 was considered as statistically significant.

**Results:**

Among 173 otitis media patients participated in the study; majority, 102(63%) were pediatrics, out of which 72 (41.61%) were in the age group of less than 4 years. Ear infection was bilateral in 39 (22.54%) and chronic in 100 (57.8%) of the patients. Pathogens were isolated from 160 (92.5%) of the patients with a total of 179 isolates. The predominant isolate was *Staphylococcus aureus* (30.72%) followed by *Proteus* spp. (17.89%). The result of this study showed that adult age (*p* = 0.031), rural residence (*p* = 0.005), previous history of health care visit and treatment (*p* = 0.000), upper respiratory tract infection (*p* = 0.018) and presence of cigarette smoker in the house (*p* = 0.022) had statistically significant association with chronic otitis media. Most of the isolated bacteria showed high level of resistance to ampicillin/amoxicillin (88.3%), penicillin G (79.5%) followed by trimethoprim /sulfamethoxazole (73.8%). Conversely, the majority of bacterial isolates showed moderate susceptibility to ciprofloxacin (72.9%), gentamicin (70.4%), and amikacin (69.3%). Bacterial isolates identified in this study showed trend of multiple drug resistance, majority (67%) being resistant to three or more antimicrobials.

**Conclusions:**

Majority of the bacterial isolates were multidrug resistant, hence, efforts to isolate microorganisms and determine the susceptibility pattern should be strengthened to improve the treatment outcome of otitis media instead of the usual trend of empirical treatment.

## Background

Ear infection which can be classified into otitis media (OM) and otitis externa, is a major public health concern in developing countries associated with high burden of disease and economic impact to patients, families and the health care system. It is one of the most frequently encountered illnesses in children leading to repeated outpatient department (OPD) visits in both developed and developing countries even if it can also affect the adults [1].

OM is a continuum of disorders ranging from a simple acute otitis media (AOM) to recurrent acute otitis media (RAOM), otitis media with effusion (OME) or chronic otitis media (COM) [2]. Untreated or inadequately treated OM due to either inaccurate diagnosis or inappropriate use of antibiotics leads to purulent otitis, often with perforation and further complications including RAOM, persistence of middle ear effusion which requires the insertion of drainage tube and often leads to hearing impairment, mastoiditis, meningitis, COM, brain abscess and sepsis [3, 4]. It also leads to impaired speech and language development, poor school performance and impaired social interaction [5]. Moreover, otitis externa is an infection of the outer ear canal commoner in patients who have eczema and or diabetes.

Diagnosis of OM is complicated by a lack of correlation between clinical features and responsible pathogens and drug susceptibility pattern. Additionally, routine unavailability of an othoscope in many of the health facilities particularly in the developing countries which is necessary to differentiate the different spectrum of clinical findings necessary for the case definition of OM also limits the health workers’ ability to make better diagnosis and classification of OM [6]. Ear infection can be classified in different ways depending on the duration of illness, and the type of clinical manifestation the patient is having or otoscopic findings [6]. In Ethiopia and other developing countries, one of the commonly used classification of ear infection is the one depending on the duration of illness, which classifies ear infection into acute ear infection (symptoms lasting for less than 14 days) and chronic ear infection (symptoms lasting for ≥14 days) [7, 8]. Empiric treatment of ear infection is not always appropriate since drug susceptibility patterns change overtime and empiric antibiotic therapy may not be effective at times and could contribute to development of antimicrobial resistance in the long run [9, 10]. Early, prompt and effective treatment of ear infection will significantly reduce both short and long term complications associated with ear infection and can also improve the quality of life of patients with ear infection. Thus, having up-to-date information on the etiologies responsible for ear infection and their antimicrobial susceptibility pattern is crucial. This study was done with an objective of determining the bacterial profile, antimicrobial susceptibility pattern and associated factors of ear infections among patients of all age groups visiting health institutions found in Jimma Town, Ethiopia during the study period with a complaint of ear discharge.

## Methods

### Study design, period and area

A prospective cross-sectional study was conducted at Jimma Town from February to September 2017 in five public health institutions: two hospitals (Jimma University Medical Center and Shanan Gibe Hospital) and three public health centers (Jimma, Higher-2, and Mendera Kochi Health Centers) that provide health services for the residents of Jimma Town and surrounding. All the health facilities give services to both children and adults. Majority of the patients coming to the health centers and Shanan Gibe Hospital are residents of Jimma Town whereas those coming to the Jimma University Medical Center (JUMC) could come from the town as well as the surrounding towns or rural areas.

### Study participants and data collection

We used consecutive sampling technique where we approached every patient coming with ear discharge during the study period. We used the presence of an ear discharge as an entry point since routine otoscopic examination might not be carried out in many of the health facilities even if this approach undermines the magnitude of ear discharge as we might have missed some patients who didn’t have an obvious ear discharge. Thus, the study participants were patients of all age category with a presumptive/clinical diagnosis of draining OM who were willing to give consent to participate in the study (parents’/care takers’ consent was taken in case of children). The relevant patient information was collected by trained nurses, while the swabs were collected by nurses at the health centers and by ENT doctor and GPs at the two hospitals. Otoscope and headlight were used during specimen collection at one of the health facilities (JUMC) whereas naked eyes were used at the other health facilities since these equipment were not available at these health facilities. We classified ear infection as acute if the duration of discharge was < 14 days and chronic if the duration of discharge was ≥14 days based on the classifications we use locally [7, 8]. All the relevant history and physical examinations were done by the treating health workers and the investigators just collected the necessary information for the study.

### Isolation and identification of bacteria

Ear discharge was collected under strict aseptic technique using single use commercially available sterile cotton swabs with utmost care to avoid surface contamination (i.e. first cleanse the external *ear* canal with antiseptic solution and then the pinna was pulled outward and backward to make it more straight forward for taking the ear swab, then sterile cotton swabs were gently rotated and taken out) and then immediately immersed into Amies transport media with charcoal (Oxoid, England).Within 2 h of collection, swabs were transported to the microbiology laboratory of JUMC, inoculated on blood agar, chocolate agar, and MacConkey agar. The MacConkey and blood agar plates were incubated in aerobic condition, whereas chocolate agar plate was kept in a candle jar, which was able to generate about 5–10% CO_2_. All of the inoculated media were incubated at 37 °C for 18–24 h. All organisms grown were identified according to the standard microbiological methods by using Gram staining reaction, culture characters, colony morphology, pigment production, and type of hemolysis on blood agar. Moreover, conventional biochemical tests like Catalase, Coagulase, Optochin and bacitracin test for gram-positive bacteria and Oxidase test, Triple Sugar Iron Agar (TSI) (OXOID, UK), Citrate utilization test (OXOID, UK), Urease test (OXOID, UK) and Motility Indole Lysine (MIL) [OXOID, UK]) for gram-negative bacteria were used for identification [11].

### Antimicrobial susceptibility testing

Antibiotic susceptibility testing was carried out using Kirby Bauer disc diffusion technique on Muller Hinton agar (Oxoid, England) [12]. Antibiotic discs were selected based on prescription pattern in the study area and recommendations from Clinical Laboratory Standards Institute (CLSI) and European Committee on Antimicrobial Susceptibility Testing (EUCAST) [13]. Accordingly, Penicillin G (P-10 U), Cefoxitin (FOX-30 μg), Ampicillin (AM-10 μg), Amoxicillin(AMX-10 μg), Erythromycin (E-15 μg), Clindamycin (CC-2 μg), Trimethoprim sulphamethoxazole (SXT-25 μg), Oxacillin (OX-1 μg), Amoxicillin plus clavulinic acid (Augmentin) (AmC-20/10 μg), Cefuroxime (CXM-30 μg), Ceftriaxone (CRO-30 μg), Ceftazidime (CAZ-30 μg), Gentamicin (GM-10 μg), Tobramycin (TOB-10 μg), Ciprofloxacin (CIP-5 μg), Vancomycin(V-30 μg), Amikacin (AK-30 μg), Cefepime (CFP-30 μg), and Chloramphenicol (C-30 μg) were used. All the antimicrobials used for the study were obtained from Oxoid Ltd., Basingstoke, Hampshire, UK. Reference strains of *E. coli* ATCC 25922 and *S. aureus* ATCC 25923 were used for quality control for antimicrobial susceptibility tests. The microbiologic results of each patient were returned back to the health workers treating the patients so that they can modify the treatment of the patients accordingly.

### Statistical analysis

The data was checked for completeness, coded, cleaned and entered into Epi-Data version 3.1 and then exported to and analyzed using SPSS version 22.0. Descriptive statistics such as frequency, percentage and cross tabulation were used to present the findings. Chi-square test was performed to evaluate the presence of statistically significant association and *P*-value less than 0.05 was considered as statistically significant.

## Results

A total of 173 patients with ear discharge participated in this study, out of which 109 (63%) were children < 14 years. Participants’ age ranged from 2 months to 70 years with a mean age of 12.8 years and median of 7 years. Most study subjects were in the age group of < 4 years (41.61%). Majority had unilateral 134 (77.46%), chronic 100 (57.8%) and purulent 113 (65.3%) ear discharge. Acute ear infection was more common in those < 4 years (53.8%) whereas chronic ear infection was more commonly seen in the age group of 15–44 years (34.6%). History of previous visit to health care facilities related to ear infection within 2 month period was noted among 102 (58.96%) patients and these patients had reportedly been treated with one or more antimicrobial agents, the most commonly prescribed antibiotics being amoxicillin, followed by amoxicillin-clavulanic acid (Table 1).

**Table 1:** Socio-demographic characteristics and clinical manifestations of patients with ear discharge at Jimma Town health facilities, Jimma, Southwest, Ethiopia (*N* = 173)

VARIABLES	FREQUENCY (%)
Sex	
Male	89 (51.44%)
Female	84 (48.56%)
Age in years	
0–4	72 (41.61%)
5–9	20 (11.56%)
10–14	17 (9.82%)
15–44	56 (32.36%)
> 45	8 (4.63%)
Residence	
Urban	125 (72.25%)
Rural	48 (27.75%)
Previous health care visit and treatment history	
Yes	102 (58.96%)
No	71 (41.04%)
Ear involvement	
Right	76 (43.93%)
Left	58 (33.53%)
Both	39 (22.54%)
Duration of ear discharge	
< 14 days	73 (42.2%)
≥14 days	100 (57.8%)
Discharge type	
Purulent	113 (65.32%)
Watery	25 (14.45%)
Mucoid	25 (14.45%)
Blood stained	10 (5.78%)
Odor of discharge	
Non-foul smelling	69 (39.88%)
Foul smelling	104 (60.12%)

The chi-square test indicated that adult age (*p* = 0.031), rural residence (*p* = 0.005), previous history of health care visit and treatment (*p* = 0.000), upper respiratory tract infection (*p* = 0.018) and presence of cigarette smoker in the house (*p* = 0.022) had statistically significant association with chronic ear infection rather than acute ear infection (Table 2).

**Table 2:** Variables identified as having statistically significant relationship to the type of ear infection cases at Jimma Town health facilities, Jimma, Southwest, Ethiopia, (*N* = 173)

Variable	Type of ear infection		*p*-value
	Acute ear infection	Chronic ear infection	
Age category			
0–4	39	35	0.031
5–9	6	14	
10–14	6	10	
15–44	22	33	
> 45	0	8	
Residence			
Urban	61	64	0.005
Rural	12	36	
Previous health care visit and treatment history			
Yes	27	76	0.000
No	46	24	
Upper respiratory tract infection			
Yes	22	48	0.018
No	51	52	
Smoker in the house			
Yes	5	19	0.022
No	68	81	

Among 173 of the study participants, 179 bacterial agents were isolated from 160 (92.5%) patients. *Citrobacter* spp. and Coagulase negative *Staphylococcus* were the predominant organisms isolated from patients with acute ear infection whereas *P. vulgaris*, *P. mirabilis* and *P. aeruginosa* were the predominant organisms isolated from patients with chronic ear infection. Among the culture positive swabs, 89.02% demonstrated single organisms, while the remaining 10.98% were positive for two organisms The rates of poly-microbial infection among acute and chronic ear infection cases were 12 (7.5%) and 7 (4.4%), respectively. Out of the 179 bacterial isolates, 12 different pathogenic bacterial species were identified. Among the isolated pathogens, Gram-negative bacteria 101 (56.4%) were predominant isolates than Gram-positives 78 (43.6%), with the ratio of ~ 1.3:1. The predominant bacterial isolate was *S. aureus* (30.72%) followed by *Proteus* spp*.* (17.89%), and *P. aeruginosa* (10.61%) (Table 3).

**Table 3:** Prevalence and distribution of bacterial isolates by age category, sex and type of otitis media at Jimma Town, Jimma, Southwest, Ethiopia (*N* = 179)

Variables	Number of Bacterial Species isolated												
	*S. aureus*	*CoNS*	*S. pyogenes*	*P. mirabils*	*P. vulgaris*	*P. aeruginosa*	*E. coli*	*Klebsiella* spp	*Provedincia* spp	*Citrobacter* spp	*Enterobacter* spp*.*	*M. morganii*	Total
Age category													
< 14 years	37	9	7	9	10	10	7	7	8	8	1	0	113 (63.1)
> 14 years	18	5	2	1	12	9	2	1	8	4	2	2	66 (36.9)
*p*-value	0.32	0.55	0.88	0.40	0.37	0.10	0.88	0.27	0.42	0.62	0.41	**0.02**	
Sex													
Male	28	8	5	7	12	5	4	5	7	7	2	1	91 (50.8)
Female	27	6	4	3	10	14	5	3	9	5	1	1	88 (49.2)
*P* value	0.94	0.60	0.75	0.20	0.68	**0.02**	0.70	0.48	0.57	0.57	0.57	0.98	
Type of OM													
AOM	27	7	4	1	4	6	6	2	5	8	1	0	72 (40.2)
COM	28	7	5	9	18	13	3	6	11	4	2	2	107 (59.8)
*P*-value	0.18	0.51	0.86	**0.037**	**0.018**	0.34	0.11	0.32	0.37	0.07	0.76	0.23	

Note: *CoNS* Coagulase-negative staphylococci, *OM* otitis media, *AOM* acute otitis media, *COM* chronic otitis media

Antimicrobial susceptibility test was done for 19 different types of antibiotics. From all the antimicrobials tested, ampicillin/amoxicillin showed the highest resistance rate (88.1%) followed by penicillin G (79.5%) and cotrimoxazole (75.8%). Conversely, the majority of bacterial isolates were susceptible to ciprofloxacin (72.9%), gentamicin (70.4%), and amikacin (69.3%).

Overall, gram-positive isolates revealed variable degree of resistance to the antimicrobials tested ranging from as low as 11.1% (for *S. pyrogens’* resistance to ampicillin, ceftriaxone, gentamicin and penicillin G) and as high as 92.7% (*S. aureus’* resistance to ampicillin, and penicillin G). Among gram-positive bacteria, increased level of resistance was found to Trimethoprim-sulphamethoxazole (78.2%), amoxicillin/ampicillin (76.9%) and penicillin G (79.5%) whereas relatively high sensitivity was seen to ceftazidime (66.7%), ciprofloxacin (70%), and gentamicin (79.5%). Out of 55 *S*. *aureus isolated,* 36 (65.5%) were Methicillin Sensitive *S. aureus* (MSSA) whereas the remaining 19 (34.5%) were Methicillin-Resistant *S. aureus* (MRSA). The majority (88.9%) of *S. pyogenes* isolates were sensitivity to penicillin G (Table 4).

**Table 4:** Antimicrobial resistance patterns of gram-positive bacterial isolates (*n* = 78) from suspected otitis media patients with discharge at Jimma Town, Jimma, Southwest, Ethiopia

*Bacterial isolates*	*Total No. isolates*	*Resistance pattern of antimicrobial agents (%)*														
		AMP	AMC	CRO	CIP	FOX	E	CN	PG	SXT	DA	OX	TOB	CTZ	CXM	CFT
*S. aureus*	55	92.7	34.5	34.5	27.3	34.5	58.2	23.6	92.7	80	43.6	34.5	40	34.5	34.5	34.5
*CoNS*	14	57.1	35.7	28.6	35.7	28.6	57.1	14.3	71.4	78.6	50	28.6	21.4	28.6	28.6	28.6
*S. pyogenes*	9	11.1	NA	11.1	NA	NA	33.3	11.1	11.1	66.7	33.3	NA	NA	NA	NA	NA
*Total*	78	76.9	34.8	30.8	30	33.3	55.1	20.5	79.5	78.2	43.6	33.3	36.2	33.3	33.3	33.3

Note: *CoNS* Coagulase-negative staphylococci, *AMP* ampicillin/amoxicillin, *AMC* amoxicillin clavulanic acid, *CRO* ceftriaxone, *CIP* ciprofloxacin, *FOX* cefoxitin, *E* erythromycin, *CN* gentamicin, *PG* penicillin G, *SXT* sulfamethoxazole-trimethoprim, *DA* clindamycin, *OX* oxacillin, *TOB* tobromycin, *CAZ* ceftazidime, *CXM* cefuroxime, *CFT* cefotaxime, *NA* not applicable

Among gram-negative isolates, maximum level of resistance was seen towards ampicillin (by *P. vulgaris, P. mirabilis*, *Klebsiella* spp*.*, *Providencia* spp*., Citrobacter* spp*.*, *Enterobacter* spp*.*, and *M. morganii*) and ceftriaxone (by *P. aeruginosa*), whereas better susceptibility was seen to ciprofloxacin (93.1%), amikacin (80.2%), gentamicin (80.2%) and cefepime (64.4%). *Proteus* spp*.*, the second most frequently isolated bacterium, showed increasing resistance rate to each of the following antibiotics: amoxicillin/ampicillin (100%), trimethoprim-sulphamethoxazole (65.6%) and chloramphenicol (59.4%); however, it showed high sensitivity to ciprofloxacin, gentamicin, and amikacin. *P. aeruginosa,* the third most common isolates, showed resistance rates to ceftriaxone and cefotaxime (100%), ceftazidime (71.4%) and cefepime (64.3%), but sensitive for gentamicin (85.7%), ciprofloxacin and amikacin (each with 78.6%). *E. coli* isolates, showed resistance rates of 88.9% to amoxicillin; however, was seen equally sensitive to chloramphenicol, amikacin, ceftazidime, ciprofloxacin. *Providencia* spp*.*, *Citrobacter* spp*.*, *Enterobacter* spp. and *Klebsiella* spp. were highly resistant to amoxicillin (100%) and amoxicillin/clavulanic acid (62.5–87.5%). On the other hand, *Citrobacter* spp*.* and *Enterobacter* spp*.* showed high level of sensitivity to gentamicin, ceftazidime, and amikacin (Table 5).

**Table 5:** Antimicrobial resistance profiles of gram-negative bacterial isolates (*n* = 101) from suspected otitis media patients with discharge at Jimma Town, Jimma, Southwest, Ethiopia

*Bacterial isolates*	*Total No. isolates*	*Resistance pattern of antimicrobial agents (%)*											
		AMP	AMC	CRO	CIP	C	FEP	CN	CTZ	SXT	TOB	AK	CXM
*E. coli*	9	88.9	33.3	33.3	11.1	11.1	22.2	33.3	11.1	77.8	66.7	11.1	44.4
*P. vulgaris*	22	100	59.1	36.4	0	54.5	27.3	18.2	27.3	63.6	50	18.2	59
*P. aeruginosa*	19	NA	NA	100	21.4	NA	64.3	14.3	71.4	NA	42.9	21.4	NA
*P. mirabilis*	10	100	50	50	10	70	50	30	30	90	40	30	70
*Klebsiella* spp.	8	100	62.5	37.5	0	37.5	25	37.5	25	62.5	25	12.5	62.5
*Providencia* spp.	16	100	87.5	62.5	12.5	56.3	56.3	25	56.3	81.3	56.3	18.8	68.8
*Citrobacter* spp.	12	100	58.3	33.3	0	41.7	16.7	8.3	16.7	66.7	66.7	16.7	66.7
*Enterobacter* spp.	3	100	66.7	33.3	0	66.7	33.3	0	0	0	66.7	0	66.7
*M. morganii*	2	100	50	50	0	0	0	0	0	50	0	0	0
*Total*	101	98.8	61	48.5	6.9	47.6	35.6	19.8	41.6	69.5	47.5	19.8	80.6

Note: *AMP* ampicillin/amoxicillin, *AMC* amoxicillin clavulanic acid, *CRO* ceftriaxone, *CIP* ciprofloxacin, *C* chloramphenicol, *FEP* cefepime, *CN* gentamicin, *CAZ* ceftazidime, *SXT* sulfamethoxazole-trimethoprim, *TOB* tobromycin, *AK* amikacin, *CXM* cefuroxime, *NA* not applicable

**Fig. 1:**
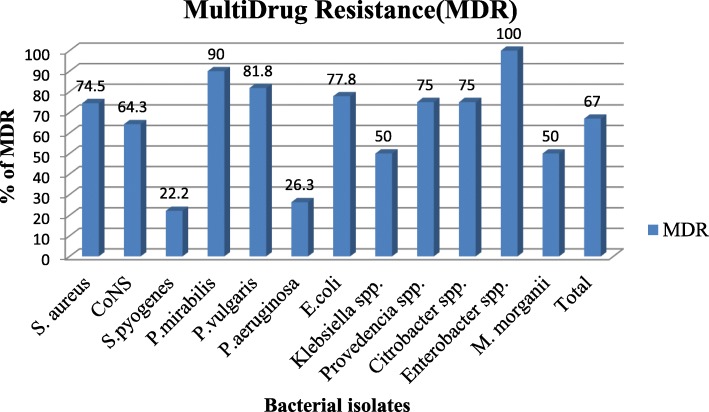
Multidrug resistance pattern of bacterial pathogens isolated from otitis media at Jimma Town, Jimma, Southwest, Ethiopia (*N* = 179)

From the total bacterial isolates, 120 (67%) had shown MDR features; *Proteus* spp. and *S. aureus* being the predominant organisms demonstrating MDR pattern (84.4 and 74.5%, respectively). The overall observed MDR rate among gram-positive and gram-negative bacterial isolates was similar (66.7 and 67.3%, respectively) (Fig. 1).

## Discussion

In the present study, the highest percentage of ear infection was found among pediatric patients (63%) and this agrees with reports from other parts of Ethiopia [3, 4, 14–17]. In addition, the majority of bacterial isolates were identified in the age group < 4 years (41.61%) which is also in line with other previous studies [4, 15, 16, 18, 19], although a study done in Dessie (Ethiopia) reported higher frequency in the age group of 16–35 years (42.4%) [20].

In this study, the majority of the patients (57.8%) had ear discharge for ≥14 days, which is in agreement with previously done studies in Ethiopia, in which COM accounted for 60–83% of the OM cases [3, 4, 21, 22]. In the present study, the bacterial isolation rate was 92.5% which was higher than previous studies reported from Hawassa, 52.1% [14], Bahir Dar 80.4% [23], Nigeria 81.9% [24], Dessie 82% [16], Wollo 83.6% [20], Gondar 89.5% [15], Dessie 89.4% [3] and lower than the reports from Mekelle 98.2% [4], and Jimma 100% [22].

In the present study, from the total bacterial isolates, gram-negative bacteria (56.4%) were slightly higher than gram-positive bacteria, which is in agreement with previous studies done in various parts of Ethiopia: Mekelle (56%), Gondar (56.4%), Bahir Dar (58.8%), Addis Ababa (60.5%), Dessie (74.2%) and (78.7%), Wollo (75.8%) and Hawassa (79.5%) [3, 4, 14–16, 18, 20, 23]. The leading isolated bacteria in this study was *S. aureus* (30.72%), followed by *Proteus* spp*.* (17.89%) and *P. aeruginosa* (10.61%), similar to reports of other investigators from Mekelle and Addis Ababa [4, 18]. Unlike our findings, *Proteus* spp. followed by *S. aureus* and *Pseudomonas* spp. were the predominant isolates reported by other researchers from different parts of Ethiopia [3, 15, 16, 20, 22] and relatively different patterns were reported from elsewhere [25–27] with *P. aeruginosa* as the main isolate followed by *S. aureus* and *Proteus* spp. The possible reasons for such variation in the bacterial profile might be attributed to the difference in climatic and geographic variation of the study sites.

In our study*,* the most prevalent organism responsible for acute ear infection was *S. aureus* (37.5%)*.* Even though, the global reports show that *H. influenzae, S. pneumoniae, and M. catarrhalis* to be the most prevalent organisms responsible for AOM [3], our findings is in agreement with reports from other African countries that indicate *S. aureus* and *S. pyogene*s were the predominant isolates [28]. The reason for this might be the differences in geographic location, prevalence of respiratory infection, coverage of pneumococcal conjugate vaccine (PCV), and possible overuse of antimicrobials that might have killed the sensitive organism and favored the drug resistant ones to be predominant, biofilm phenotypes property of the *S. aureus* and potentially other local and regional factors.

In the present study, both *Proteus* spp*.* (*P. vulgaris* (*p* = 0.018) and *P. mirabilis* (*p* = 0.037)) (84.4%) were more common among the chronic than the acute ear infections. This finding is comparable to Seid et al. from Dessie [3] and Muleta et al. from Jimma [22] who reported rates of 85.4 and 74.5%, respectively and contradicts with Wasihun and Zemene’s from Mekele [4] who reported that *P. mirabilis* was seen in 63% of COM and *P. vulgaris* in 57% of AOM. The possible reasons for this might be *Proteus* spp*.* were common isolates in patients presenting lately (2 months after onset of ear discharge), as a result patients with discharging ears may not notice immediately for early diagnosis or the antibiotic treatment was not effective [29].

In this study, those with history of previous health care visit and treatments (58.96%) showed a significant association with chronic ear infection (*p* = 0.000), which is similar to Wasihun’s and Zemene’s reports [4]. This could probably be due to failure of empiric treatment of AOM. Thus, it might be wise to take a swab if the patient has ear discharge, then begin treating the patient with topical +/− systemic antibiotics according to empirical guidance available. In the meantime, proper etiology based diagnosis should be in place and once the organism is known, appropriate management of ear infection becomes effective. This in turn facilitates the rational use of antibiotics based on recent and local data in our health facilities in order to reduce sequales associated with ear infection [1]. In this study, the isolated bacteria showed highest rate of resistance to the different antibiotics commonly used for OM treatment which is in line with earlier reports from different parts of Ethiopia [3, 4, 16, 20] and a good overall antimicrobial susceptibility pattern (> 70%) was seen to gentamicin and ciprofloxacin which is also in line with other studies conducted in Ethiopia [3, 4, 16, 20, 23] and in other countries asuch as Ardebil [30], Iraq [31], Nepal [32], India [33], and Jordan [34]). In contrast to these reports, gentamicin and ciprofloxacin were reported as ineffective from a study conducted in Nigeria [24].

In the present study, 34.5% of *S. aureus* were MRSA, similar to a report by Hailu et al. (34.6%) from Bahir Dar [23]. On the other hand*, S. aureus* exhibited high levels of resistance to ciprofloxacin (27.3%) which is significantly higher compared with other reports which showed resistance rates as high as 21% [3, 4, 15, 16, 23]. Many of the isolates showed high levels of sensitivity to gentamicin, which is consistent with other reports [14–16]. Moreover, the observed high level of resistance for clindamycin (43.6%), erythromycin (58.2%) and trimethoprim-sulphamethoxazole (80%) is also higher than other studies [3, 4, 15, 16, 22].

Overall, 67% of the bacterial isolates from this study were characterized as MDR pathogenic bacteria. The reason for this might be linked to a prescription of antibiotics without laboratory guidance, purchasing of drugs without proper prescription (self-medication) in the local pharmacies and drug stores, misuse of antibiotics, indiscriminate use of antibiotics including animal husbandry, inappropriate prescribing habits and an over-zealous desire to treat every infection using antibacterial agents. Moreover, biofilm bacterial properties of common isolates, unavailability of bacterial culture facilities and poor infection prevention and control practices may be some of the different factors that can contribute to the development of MDR among these isolates.

The limitations of this study include the fact that we did not include the OME and OM without discharge as well as the fact that we did not try to isolate strict anaerobic bacteria and fungi which might also be the possible causative agents for OM. Additionally, we didn’t intend to differentiate OM from otitis externa.

## Conclusion and recommendation

*S. aureus*, *Proteus* spp. and *P. aeruginosa* were the three predominant bacterial isolates from patients with ear discharge. The profile and predominant bacteria isolated highlights the need for continuous surveillance and reporting of the microbiology of ear infection in our local community in order to guide clinicians use the appropriate antimicrobials towards the incriminated etiologies. The pathogens which have been isolated from ear discharge have shown a high level of antibiotics resistance and MDR features in the study area. Almost all the isolated bacteria showed a considerable level of resistance particularly to the commonly used antibiotics like ampicillin, amoxicillin, amoxicillin/clavulanic acid, and trimethoprim-sulfamethoxazole. Ciprofloxacin, amikacin and gentamicin are effective against all the bacterial isolates. In general, the result of this study revealed that antibiotic-resistant bacteria recovered from patients with ear infection are alarmingly increasing in Jimma area and becoming a major public health problem in the management of patients with a middle ear infection. Due to the increase in resistance to antibiotics one can advocate to swab the ear first and wait for the result especially if the patient has already had treatment as shown by our data which is often the case. We strongly recommend nationwide antimicrobial surveillance to make the right recommendation of antibiotics along with strict adherence to antibiotic use policy to reduce the spread of drug-resistant microbes and associated complications in the country. Hence, parallel to empiric treatment of ear infection, execution of culture and antimicrobial susceptibility test shall be taken as routine and mandatory practice in order to appropriately manage ear infection, reduce associated complications (individual, household, and health system) and reduce emergence of drug resistance in the community. Moreover, in the new cases we recommend taking swab before starting a regime based treatment in case there is already resistance. Further researches are needed to identify high resistance strains and characterization of resistance strains using molecular techniques.

## Abbreviations

AOM: Acute otitis media
ATCC: American Type Culture Collection
CLSI: Clinical Laboratory Standards Institute
COM: Chronic otitis media
EUCAST: European Committee on Antimicrobial Susceptibility Testing
MDR: Multidrug resistance
MRSA: Methicillin-Resistant *S. aureus*
MSSA: Methicillin Sensitive *S. aureus*
OM: Otitis media
OME: Otitis media with effusion
RAOM: Recurrent acute otitis media
